# The* CYBA* Gene ^⁎^49A>G Polymorphism (rs7195830) Is Associated with Hypertension in Patients with Coronary Artery Disease

**DOI:** 10.1155/2016/1539671

**Published:** 2016-05-24

**Authors:** Tomasz Nowak, Paweł Niemiec, Sylwia Górczyńska-Kosiorz, Anna Balcerzyk, Tomasz Iwanicki, Jolanta Krauze, Wladyslaw Grzeszczak, Anna Ochalska-Tyka, Joanna Iwanicka, Iwona Zak

**Affiliations:** ^1^School of Health Sciences in Katowice, Department of Biochemistry and Medical Genetics, Medical University of Silesia, Medyków Street 18, 40-752 Katowice, Poland; ^2^School of Medicine and Division of Dentistry in Zabrze, Department of Internal Medicine, Diabetes and Nephrology, Medical University of Silesia, 3 Maja Street 13-18, 41-800 Zabrze, Poland; ^3^1st Department of Cardiac Surgery/2nd Department of Cardiology, American Heart of Poland, S. A. Armii Krajowej Street 101, 43-316 Bielsko-Biala, Poland; ^4^Regional Centre of Blood Donation and Blood Treatment in Raciborz, Sienkiewicza Street 3, 47-400 Raciborz, Poland

## Abstract

*Purpose*. Single nucleotide polymorphisms of the* CYBA* gene may modify the risk of coronary artery disease (CAD). The aim of the present study was to investigate whether the ^⁎^49A>G (rs7195830) polymorphism is associated with CAD.* Materials and Methods*.* CYBA* gene ^⁎^49A>G polymorphism was determined in 481 subjects: 242 patients with premature CAD and 239 age and sex matched controls using the fluorescently labeled allele-specific oligonucleotides method.* Results*. The frequency of the ^⁎^49G allele carrier state was significantly higher in patients than in controls (84.8% versus 76.6%, resp., *P* = 0.020), as well as the frequency of the ^⁎^49G allele (62.2% versus 54.0%, *P* = 0.009). Both factors were associated with CAD in the analyzed population (OR = 1.70, 95% CI: 1.04–2.76 for GG+AG versus AA and OR = 1.40, 95% CI: 1.08–1.83 for ^⁎^49G versus  ^⁎^49A). Carrier state of the ^⁎^49G allele was a stronger and independent risk factor for CAD among women (OR = 4.35, 95% CI: 1.50–13.20, *P* = 0.002), as well as the ^⁎^49G allele (OR = 2.25, 95% CI: 1.34–3.77, *P* = 0.001). The ^⁎^49G allele carrier state was also associated with left ventricular hypertrophy in patients with coronary artery disease (*P* = 0.015).* Conclusion*. The* CYBA* gene ^⁎^49A>G polymorphism modifies the risk of coronary artery disease.

## 1. Introduction

NADPH oxidases (nicotinamide adenine dinucleotide phosphate oxidases) are one of the main sources of reactive oxygen species (ROS) in human body and especially in vasculature. NADPH oxidases are expressed in endothelium, vascular smooth muscle cells, fibroblasts, cardiomyocytes, and mainly phagocytes. Free radicals produced by NADPH oxidases are involved in the regulation of ROS-dependent intracellular signaling pathways, affecting expression of many classes of genes. In physiological conditions, ROS can stimulate growth, differentiation, migration, division, and apoptosis of cells of the vessel wall, to regulate vascular remodeling processes [1–5]. When synthesized in excessive levels, ROS may intensify the progress and development of atherosclerosis. Many data suggest the role of NADPH oxidases in the pathogenesis of cardiovascular disorders with atherosclerotic background, including coronary artery disease (CAD) [3, 6–9].

NADPH oxidases are multisubunit enzymatic complexes, which produce superoxide anion from molecular oxygen [6, 10]. Their active complexes are composed of NOX (NADPH oxidase) catalytic protein and many auxiliary subunits. There are few types of NOX proteins, namely, NOX2 (gp91phox) and its homologues (NOX1, NOX3–5). NOX2-based oxidases have the most important role in vascular superoxide production. Membrane-bound p22phox and cytoplasmic p47phox, Noxo1 (p47phox homologue), p67, Noxa1 (p67phox homologue), p40phox, and Rac GTP-ases are additional subunits of NADPH oxidases active complexes. p22phox and NOX form a heterodimer named cytochrome b_558_. During assembly of an active complex, cytosolic subunits migrate to the membrane-bound cytochrome b_558_ which becomes activated and generates superoxide anions [11–17].

The p22phox is common element of NADPH oxidases complexes based on NOX1–NOX4 proteins [9]. It stabilizes the NOX-p22phox heterodimer formation and acts as a link between NOX and cytoplasmic subunits. p22phox is encoded by* CYBA* (cytochrome b_245_ alpha) gene (16q24). Opposite to genes encoding other subunits of NADPH oxidases, the* CYBA* gene is characterized by a relatively high polymorphic variability. It is probably due to the location of the* CYBA* gene, near a telomeric end of chromosome 16, and is a result of higher frequency of recombination than in loci of genes for other components of NADPH oxidases [8, 9].

In our previous studies, we demonstrated that single nucleotide polymorphisms (SNPs) of the* CYBA* gene may increase the risk of CAD in the Polish population [18] or intensify the effects caused by influence of traditional risk factors like cigarette smoking [18–20], hypercholesterolemia [19, 20], or overweight/obesity [18]. The current study presents results from the next step of research on* CYBA* gene polymorphisms in the context of CAD and focuses on ^*∗*^49A>G (rs7195830) SNP. The ^*∗*^49A>G (NC_000016.9:g.88709712A>G, NM_000101.2:c. ^*∗*^49T>C) polymorphism lies in the 3′ UTR (3′ untranslated region) of the* CYBA* gene. To date, very little is known about this polymorphism and its potential functionality. Lack of previous studies with regard to its association with coronary artery disease prompted us to undertake such research. The aim of the present study was to investigate whether the ^*∗*^49A>G polymorphism shows an association with CAD and its clinical phenotype and whether it modifies the risk of disease associated with exposure to the traditional risk factors.

## 2. Materials and Methods

### 2.1. Subjects

We studied 481 Polish Caucasians, inhabitants of Upper Silesia. There were 242 patients with angiographically documented premature coronary artery disease in the CAD group (69 females and 173 males), aged 27–55 years (mean 44.79 ± 5.94). The second group included 239 blood donors (BD) matched by age and gender, with no signs of CAD and with negative familial history of CAD as inclusion criteria. CAD subjects were classified by the same cardiologist from (1) patients admitted to the 1st Department and Clinic of Cardiology at the Upper Silesian Center of Cardiology in Katowice and (2) patients admitted to the 1st Department of Cardiac Surgery at the Upper Silesian Center of Cardiology in Katowice. Blood donors were used as a control group. They were recruited from the Regional Centers of Blood Donation and Blood Treatment in Katowice and Raciborz. Blood was collected only from subjects with systolic blood pressure (SBP) < 140 mmHg and diastolic blood pressure (DBP) < 90 mmHg on the day of blood collection, according to recommendations of the Polish Centers of Blood Donation and Blood Treatment.

The criteria of inclusion and exclusion to the study as well as criteria for coronary artery disease, myocardial infarction (MI), and common risk factors of CAD were described previously [20].

The study was approved by the Ethics Committee of the Medical University of Silesia in Katowice. All submitted cases signed an informed consent prior to the study.

### 2.2. Methods

#### 2.2.1. Serum Lipids Measurement

Serum lipid parameters like triglycerides (TG), total cholesterol (TC), and HDL cholesterol (HDL-C) were measured using colorimetric methods (Analco, Warsaw, Poland). The levels of LDL cholesterol (LDL-C) were calculated using Friedewald formula [21] only in subjects with TG levels < 4.4 mmol/L.

#### 2.2.2. DNA Extraction and Genotyping

Genomic DNA was extracted using the MasterPure genomic DNA purification kit (Epicentre Technologies, Madison, USA). The* CYBA* gene ^*∗*^49A>G polymorphism (rs7195830) was genotyped using the TaqMan® Predesigned SNP Genotyping Assay (Applied Biosystems, Foster City, USA). Primer-probe sets were designed and prepared by the manufacturer. 7300 Real-Time PCR System (Applied Biosystems, Foster City, USA) was used to genotyping the rs7195830 polymorphism. Correctness of the genotyping was verified by regenotyping 15% of the samples and the repeatability was 100%.

#### 2.2.3. Statistical Analysis

*Statistica 10.0* software (STATSOFT, Tulsa, USA) was used in statistical analyses. Normality of distribution of quantitative data was assessed by the Shapiro-Wilk test and then a comparison of data was performed by Student's* t*-test (variables with normal distribution) or the Mann-Whitney* U* test (variables with nonnormal distribution).

The *χ* ^2^ test was used to test the Hardy-Weinberg equilibrium in all groups. Genotypes and alleles frequencies were also compared by *χ* ^2^ test as well as the presence of possible associations between genotype variants of the ^*∗*^49A>G polymorphism and characteristics of clinical phenotype of CAD. Odds ratios (OR) and their 95% confidence intervals (CI) were estimated using a univariate analysis. In univariate analyses statistical significance was accepted at *P* < 0.05. Multiple logistic regression analysis was performed after adjustment for age, gender, and traditional risk factors of CAD. Only variables with *P* values of less than 0.1 in univariate analysis were classified to the multiple logistic regression model. Risk ratio (RR) values with 95% CI were used when the number of subjects in any of subgroups was 0.* Epi Info™ 7.1.1.0* (Centers for Disease Control and Prevention, Atlanta, USA) was used to compute the effective sample size and statistical power.

To determine possible interactions between* CYBA* genotypes and traditional risk factors of CAD (synergism/antagonism), the 4 × 2 table approach was used. Multiplicative synergy indices (SIMs) were calculated on the basis of OR values taken from 4 × 2 table comparisons [22]. 95% confidence intervals for SIMs were calculated using the script described by Cortina-Borja et al. [23]. Additive interactions were analyzed independently by a *χ* ^2^ test using ORs as measures of interaction.

## 3. Results

### 3.1. Clinical Characteristics and Traditional Risk Factors of CAD

There were 72.5% cases who had suffered from MI in the CAD group. 65.8% of patients had multivessel disease (stenoses in at least two coronary vessels). Critical stenoses (>90%) in coronary vessels were diagnosed in 66.9% patients. 12% of patients with CAD suffered from a left ventricular hypertrophy (LVH). Other clinical and biochemical parameters of patients and controls are shown in Table 1. CAD patients had increased levels of TC, LDL-C, and TG and a higher BMI. HDL-C levels were significantly lower in CAD patients. Hypertension, cigarette smoking, and diabetes mellitus were significantly more frequent in CAD subjects (Table 1).

### 3.2. Association of the CYBA Gene ^⁎^49A>G Polymorphism with CAD

Genotype frequencies of the* CYBA* gene ^*∗*^49A>G polymorphism were compatible with the Hardy-Weinberg equilibrium in CAD group and controls (*P* = 0.81, *P* = 0.35, resp.), as well as in subgroups divided by gender (*P* = 0.86 in CAD females, *P* = 0.58 in CAD males and *P* = 0.63 in BD females, *P* = 0.63 in BD males).

The frequency of the ^*∗*^49G allele carriers (GG+AG genotypes) was significantly higher in patients than in controls as well as the frequency of the ^*∗*^49G allele (Table 2). The GG genotype was also more frequent in the CAD group than in the controls; however the difference obtained in univariate model lied on the border of statistical significance (*P* = 0.057). Both the ^*∗*^49G allele carrier state and GG homozygosity were associated with premature CAD in multivariate model, after adjustment for sex, age, TC, LDL-C, HDL-C, TG, BMI, diabetes mellitus, cigarette smoking status, hypertension, and familial history of coronary artery disease (Table 2).

Further analyses showed that the differences in the frequencies of genotypes and alleles of the ^*∗*^49A>G* CYBA* polymorphism were more significant in female subgroups of CAD patients and blood donors (Table 3). The ^*∗*^49G allele carrier state and GG homozygosity were associated with premature CAD in multivariate model, also in this case (Table 3). These associations were not observed in male subgroups (data not shown).

### 3.3. The CYBA Gene ^⁎^49A>G Polymorphism and Traditional Risk Factors of CAD

The presence of possible interactions (synergy/antagonism, additive and/or multiplicative effects) between genotype variants of the* CYBA* gene ^*∗*^49A>G polymorphism and traditional risk factors of CAD (cigarette smoking, BMI, elevated levels of TC, and LDL) was checked. We did not find any statistically significant interactions between ^*∗*^49A>G polymorphism genotypes and traditional risk factors of CAD (data not shown).

We also checked whether the ^*∗*^49A>G polymorphic variants are associated with concomitant phenotypes of CAD like hypertension, dyslipidemias, and diabetes mellitus, using case-control model. We found an association between rs7195830 SNP variants and hypertension analysing subgroups of hypertensive CAD patients (*n* = 137) and nonhypertensive blood donors (*n* = 232). The frequencies of GG genotype, ^*∗*^49G allele carriers (subjects with GG+AG genotypes), and the ^*∗*^49G allele were significantly higher in hypertensive CAD patients than in controls (Table 4). There were no statistically significant differences in alleles and genotypes distribution between nonhypertensive CAD patients and nonhypertensive blood donors (data not shown).

The presence of possible associations between variants of the ^*∗*^49A>G polymorphism and characteristics of clinical phenotype of CAD was also tested. The ^*∗*^49G allele was present in 100% of CAD patients with left ventricular hypertrophy and its frequency in patients without LVH was 82.6%. This difference was statistically significant (Table 5). There was no association between genotype variants of the ^*∗*^49A>G polymorphism and MI or the severity of atherosclerosis estimated on the basis of the number of coronary stenoses or critical arterial occlusions observed during a coronary angiography (data not shown).

## 4. Discussion

The* CYBA* gene encodes p22phox, a subunit of NADPH oxidases, enzymes critical in vascular production of superoxide anion. In the current work the ^*∗*^49A>G* CYBA* gene polymorphism was analyzed in the context of coronary artery disease in the classic case-control model. The influence of the ^*∗*^49A>G SNP on CAD risk and possible interactions with traditional risk factors of CAD were searched. We demonstrated statistically significant differences in genotype and allele frequencies of the ^*∗*^49A>G polymorphism between analyzed groups. ^*∗*^49G allele carriers were more frequent in CAD patients than in the control group and ^*∗*^49G allele carrier state was associated with CAD. We also showed that carrier state of the ^*∗*^49G allele is a stronger and independent risk factor for CAD among women. Present work also demonstrated that the ^*∗*^49G allele carrier state is associated with left ventricular hypertrophy in patients with coronary artery disease.

This last observation prompted us to check whether the rs7195830 polymorphism was associated with hypertension, as the left ventricular hypertrophy is most often the result of high blood pressure. We observed that the GG genotype, ^*∗*^49G allele, and its carrier state were independent risk factors of hypertension, when we compared extracted subgroups of CAD patients (only hypertensive subjects) and blood donors (only nonhypertensive controls). This effect was stronger than that observed between the analyzed polymorphism and CAD, which suggests that the rs7195830 is a risk factor for hypertension rather than for CAD.

Hypertension is one of the main causes of left ventricular hypertrophy. It seems that there is positive feedback between hypertension-induced myocardial hypertrophy and NADPH oxidases. On the one hand NADPH oxidases of the neuronal, renal, and vascular systems are strongly implicated in pathological signaling leading to hypertension (discussed in Datla and Griendling [24]). It is well documented that NOX are a primary source of superoxide in angiotensin II-induced neuronal and vascular activity and superoxide induces vascular dysfunction in hypertension by its well-described interaction with NO. On the other hand, hypertension is known activator of NADPH oxidases. NADPH oxidases may be involved in cardiac hypertrophy, because their expression and activity are upregulated by pressure overload, in both cardiomyocytes and endothelial cells of rodents [25, 26].

Until today, the* CYBA* gene ^*∗*^49A>G polymorphism was not related to hypertension; however, other* CYBA* gene SNPs, namely, -930A>G and -675A>T, were previously associated with this phenotype [3, 8, 27, 28]. It was also shown that the T allele of the 214C>T polymorphism is associated with higher left ventricular mass in Brazilian hypertensive patients [29].

Lack of functional studies and insufficient number of clinical trials greatly hinder possibility of comprehensive interpretation of the results of our work. Although the function of the ^*∗*^49A>G polymorphism is not yet known, Fan et al. [30] suggested that the rs7195830 lies in a miRNA binding region of the 3′ UTR of* CYBA*. Because binding of miRNA to mRNA is essential for mRNA level regulation and protein expression, it is possible that allelic variants of the ^*∗*^49A>G polymorphism may differentially affect miRNA-mediated efficiency and a rate of these processes. Although this is only a hypothesis, the neighboring polymorphism of the 3′ UTR of the* CYBA* gene, namely, rs1049255 (^*∗*^24G>A), lies within the miRNA binding site [31]. The ^*∗*^24A allele increases the potential of hybridization between* CYBA* mRNA and miRNA (*miR-320a*) so lower levels of* CYBA* expression and NOX activity observed in ^*∗*^24A allele may result from enhanced regulation of miRNA [31]. It is possible that an association between ^*∗*^49G allele and CAD observed in the present work is a consequence of the interactions between these two 3′ UTR polymorphisms, especially as the distance between the ^*∗*^24G>A and ^*∗*^49A>G SNPs is only 25 base pairs. It should be noted, however, that we did not observe preferential coexistence of alleles of both polymorphisms and they did not create haplotype blocks in our studied groups (data not shown). In our previous study, however, we documented that the ^*∗*^24G allele influences predispositions to CAD through interactions with cigarette smoking and hypercholesterolemia with no direct association with the disease [20].

To date the ^*∗*^49A>G* CYBA* polymorphism was analyzed only in the aspects of artery elasticity [32], chronic kidney disease [30], and cervical cancer progression [33]. In the study analyzing large and small artery elasticity of the apparently healthy Chinese individuals the GG and AG genotypes had higher small artery elasticity compared with the AA genotype (*P* = 0.049), in physically active participants. There was no such association in less physically active participants [32]. Next work determined whether the ^*∗*^49A>G SNP influences the estimated glomerular filtration rate (eGFR) and its role in the pathogenesis of chronic kidney disease in Chinese population [30]. The results indicated that the AA homozygosity of the ^*∗*^49A>G polymorphism was associated with significantly lower eGFRs (*P* = 0.031) compared with the ^*∗*^49G allele carrier state. The ^*∗*^49A allele carrier state was associated with an increased risk of intraepithelial neoplasia grade 3 or cervical cancer, in subjects with papillomavirus infections [33].

As seen, it is difficult to relate our findings to the results of the previous studies, mainly due to the difference of studied phenotypes/disease entities and ethnic differences between the studied populations. The latter factor appears to be particularly important. In some cases, the presence of differences in allele and genotype frequencies of SNPs results in contradictory findings of association studies from populations with different ethnicity. The results of the meta-analyses of another often tested* CYBA* polymorphism, namely, 242C>T (rs4673), can be an example of the foregoing. In Caucasians, the TT genotype was associated with an increased risk of ischemic cerebrovascular disease (OR = 2.13, 95% CI: 1.06–4.26, *P* = 0.034) [34] and marginally with CAD (OR = 1.22, 95% CI: 0.99–1.49, *P* = 0.06) [35]. In Asians, the T allele has a protective effect to coronary artery disease [36, 37] and ischemic cerebrovascular disease [34].

Summing up the results of our current work, carrier state of the ^*∗*^49G allele of the* CYBA* gene is associated with coronary artery disease, hypertension, and left ventricular hypertrophy in patients with coronary artery disease. The ^*∗*^49A>G polymorphism probably does not modify the risk of CAD through interactions with known, traditional risk factors, unlike other* CYBA* gene polymorphisms [18–20]. The results of the current work tend to indicate that the ^*∗*^49A>G polymorphism may be particularly strong risk factor in females. Since neither current nor previous studies explain the role of the ^*∗*^49A>G polymorphism and its relationship with hypertension and atherosclerotic CAD, further functional researches and population surveys are needed.

## Figures and Tables

**Table 1:** Biochemical and clinical characteristics of analyzed groups.

Characteristic	CAD *N* = 242	BD *N* = 239	Statistics results
	Mean ± SD	Mean ± SD	*P*
Age (years)	44.79 ± 5.94	43.62 ± 6.46	0.92
TC (mmol/L)	5.81 ± 1.34	5.06 ± 1.21	<0.000
LDL-C (mmol/L)	4.03 ± 1.20	3.11 ± 1.17	<0.000
HDL-C (mmol/L)	1.11 ± 0.37	1.44 ± 0.56	<0.000
TG (mmol/L)	1.89 ± 0.97	1.39 ± 0.73	<0.000
BMI	27.24 ± 4.16	26.08 ± 3.83	0.001

	% (*n*)	% (*n*)	OR (95% CI), *P* univariate analysis

Male gender	70.6 (173)	71.0 (174)	0.98 (0.65–1.47), 0.92
TC ≥ 5 mmol/L	70.0 (168)	47.9 (117)	2.53 (1.71–3.75), <0.000
LDL ≥ 3 mmol/L	77.6 (184)	50.4 (123)	3.42 (2.26–5.17), <0.000
HDL-C ≤ 1 mmol/L ♂	48.1 (114)	24.6 (60)	2.84 (1.90–4.27), <0.000
≤ 1.2 mmol/L ♀			
TG ≥ 1.7 mmol/L	53.3 (128)	27.0 (66)	3.14 (2.11–4.68), <0.000
BMI ≥ 25	70.7 (152)	56.1 (129)	1.89 (1.25–2.85), 0.001
Cigarette smoking	60.3 (146)	27.8 (68)	3.94 (2.64–5.87), <0.000
Hypertension	58.2 (137)	2.9 (7)	46.00 (19.94–111.51), <0.000
Diabetes mellitus	8.7 (21)	0 (0)	2.08 (1.89–2.29), <0.000^1^
Familial history of CAD	36.0 (87)	0 (0)	2.54 (2.25–2.87), <0.000^1^

^1^Risk ratio values (95% CI), univariate analysis.

CAD: coronary artery disease patients, BD: blood donors, and OR: odds ratio.

**Table 2:** The frequency of genotypes and alleles of the *CYBA *gene ^*∗*^49A>G polymorphism in the groups of patients (CAD) and blood donors (BD).

Genotype, allele	CAD (*N* = 242) % (*n*)	BD (*N* = 239) % (*n*)	Versus	OR (95% CI), *P* univariate analysis	Power (%) 95% CL
AA	15.2 (37)	23.4 (56)	GG+AG	0.59 (0.36–0.96), 0.020^1^	61
AG	45.1 (109)	45.2 (108)	—	—	—
GG	39.7 (96)	31.4 (75)	AA+AG	1.44 (0.97–2.13), 0.057^2^	47
AA+AG	60.3 (146)	68.6 (164)	GG	0.70 (0.47–1.03), 0.057^3^	47
GG+AG	84.8 (205)	76.6 (183)	AA	1.70 (1.04–2.76), 0.020^4^	61
^*∗*^49A	37.8 (183)	46.0 (220)	^*∗*^49G	0.71 (0.55–0.93), 0.009	72
^*∗*^49G	62.2 (301)	54.0 (258)	^*∗*^49A	1.40 (1.08–1.83), 0.009	72

The results of multivariate analysis after adjustment for sex, age, TC, LDL-chol, HDL-chol, TG, BMI, diabetes mellitus, cigarette smoking status, hypertension, and familial history of CAD.

^1^OR = 0.45, 95% CI; 0.25–0.83, *P *= 0.011.

^2^OR = 2.05, 95% CI; 1.24–3.38, *P* = 0.005.

^3^OR = 0.48, 95% CI; 0.30–0.81, *P* = 0.005.

^4^OR = 2.19, 95% CI; 1.20–3.99, *P* = 0.011.

CAD: coronary artery disease patients, BD: blood donors, OR: odds ratio, CI: confidence interval, and CL: confidence level.

**Table 3:** The frequency of genotypes and alleles of the *CYBA *gene ^*∗*^49A>G polymorphism in the female subgroups of patients (CAD) and blood donors (BD).

Genotype, allele	CAD (*N* = 70) % (*n*)	BD (*N* = 69) % (*n*)	Versus	OR (95%CI), *P* univariate analysis	Power (%) 95% CL
AA	8.6 (6)	29.0 (20)	GG+AG	0.23 (0.08–0.67), 0.002^1^	87
AG	45.7 (32)	43.5 (30)	—	—	—
GG	45.7 (32)	27.5 (19)	AA+AG	2.22 (1.03–4.79), 0.020^2^	60
AA+AG	54.3 (38)	72.5 (50)	GG	0.45 (0.21–0.97), 0.020^3^	60
GG+AG	91.4 (64)	71.0 (49)	AA	4.35 (1.50–13.20), 0.002^4^	87
^*∗*^49A	31.4 (44)	50.7 (70)	^*∗*^49G	0.45 (0.26–0.75), 0.001	86
^*∗*^49G	68.6 (96)	49.3 (68)	^*∗*^49A	2.25 (1.34–3.77), 0.001	86

The results of multivariate analysis after adjustment for sex, age, TC, LDL-chol, HDL-chol, TG, BMI, diabetes mellitus, cigarette smoking status, hypertension, and familial history of CAD.

^1^OR = 0.14, 95% CI; 0.03–0.66, *P *= 0.012.

^2^OR = 4.87, 95% CI; 1.41–16.78, *P *= 0.011.

^3^OR = 0.21, 95% CI; 0.06–0.71, *P *= 0.011.

^4^OR = 6.99, 95% CI; 1.52–32.16, *P* = 0.012.

CAD: coronary artery disease patients, BD: blood donors, OR; odds ratio, CI: confidence interval, and CL: confidence level.

**Table 4:** The frequency of genotypes and alleles of the *CYBA *gene ^*∗*^49A>G polymorphism in subgroups of hypertensive CAD patients (CAD) and nonhypertensive blood donors (BD).

Genotype, allele	CAD % (*n*)	BD % (*n*)	Versus	OR (95% CI), *P* univariate analysis	Power (%) 95% CL
AA	13.1 (18)	22.7 (49)	GG+AG	0.48 (0.27–0.85), 0.011^1^	73
AG	43.1 (59)	45.4 (98)	—	—	—
GG	43.8 (60)	31.9 (69)	AA+AG	1.66 (1.08–2.57), 0.022^2^	62
AA+AG	56.2 (77)	68.1 (147)	GG	0.60 (0.39–0.93), 0.022^3^	62
GG+AG	86.9 (119)	77.3 (167)	AA	2.10 (1.18–3.76), 0.011^4^	73
^*∗*^49A	34.7 (95)	45.4 (196)	^*∗*^49G	0.62 (0.45–0.84), 0.002	60
^*∗*^49G	65.3 (179)	54.6 (236)	^*∗*^49A	1.61 (1.18–2.20), 0.002	60

The results of multivariate analysis after adjustment for sex, age, TC, LDL-chol, HDL-chol, TG, BMI, diabetes mellitus, cigarette smoking status, and familial history of CAD.

^1^OR = 0.47, 95 % CI; 0.25–0.91, *P* = 0.024.

^2^OR = 1.90, 95 % CI; 1.14–3.15, *P* = 0.012.

^3^OR = 0.53, 95 % CI; 0.32–0.87, *P* = 0.012.

^4^OR = 2.10, 95 % CI; 1.10–4.01, *P* = 0.024.

CAD: coronary artery disease patients, BD: blood donors, OR: odds ratio, CI; confidence interval, and CL: confidence level.

**Table 5:** The frequency of genotypes and alleles of the *CYBA *gene ^*∗*^49A>G polymorphism in CAD patients with left ventricular hypertrophy (+LVH) and without LVH (−LVH).

Genotype, allele	+LVH % (*n*)	−LVH % (*n*)	Versus	*χ* ^2^, *P*	Power (%) 95% CL
AA	0 (0)	17.4 (37)	GG+AG	5.95, 0.015	70
AG	51.7 (15)	44.1 (94)	—	—	—
GG	48.3 (14)	38.5 (82)	AA+AG	NS (*P *= 0.31)	—
AA+AG	51.7 (15)	61.5 (131)	GG	NS (*P *= 0.31)	—
GG+AG	100.0 (29)	82.6 (176)	AA	5.95, 0.015	70

+LVH: presence of left ventricular hypertrophy, −LVH: lack of left ventricular hypertrophy, CL: confidence level, and NS: not statistically significant.

## References

[1] Cai H., Harrison D. G. (2000). Endothelial dysfunction in cardiovascular diseases: the role of oxidant stress. *Circulation Research*.

[2] Sirker A., Zhang M., Shah A. M. (2011). NADPH oxidases in cardiovascular disease: insights from in vivo models and clinical studies. *Basic Research in Cardiology*.

[3] Moreno M. U., San José G., Fortuño A. (2007). A novel CYBA variant, the -675A/T polymorphism, is associated with essential hypertension. *Journal of Hypertension*.

[4] Griendling K. K., Sorescu D., Ushio-Fukai M. (2000). NAD(P)H oxidase: role in cardiovascular biology and disease. *Circulation Research*.

[5] Lassègue B., Clempus R. E. (2003). Vascular NAD(P)H oxidases: specific features, expression, and regulation. *American Journal of Physiology—Regulatory Integrative and Comparative Physiology*.

[6] Sumimoto H., Miyano K., Takeya R. (2005). Molecular composition and regulation of the Nox family NAD(P)H oxidases. *Biochemical and Biophysical Research Communications*.

[7] Niemiec P., Żak I. (2005). Naczyniowe oksydazy NAD(P)H—znaczenie w patogenezie miażdżycy. *Postępy Biochemii*.

[8] Moreno M. U., San José G., Orbe J. (2003). Preliminary characterisation of the promoter of the human p22^phox^ gene: identification of a new polymorphism associated with hypertension. *FEBS Letters*.

[9] San José G., Fortuño A., Beloqui Ó., Díez J., Zalba G. (2008). NADPH oxidase CYBA polymorphisms, oxidative stress and cardiovascular diseases. *Clinical Science*.

[10] Davis A. R., Mascolo P. L., Bunger P. L., Sipes K. M., Quinn M. T. (1998). Cloning and sequencing of the bovine flavocytochrome b subunit proteins, gp91-phox and p22-phox: comparison with other known flavocytochrome b sequences. *Journal of Leukocyte Biology*.

[11] Dworakowski R., Alom-Ruiz S. P., Shah A. M. (2008). NADPH oxidase-derived reactive oxygen species in the regulation of endothelial phenotype. *Pharmacological Reports*.

[12] Dikalov S. I., Dikalova A. E., Bikineyeva A. T., Schmidt H. H. H. W., Harrison D. G., Griendling K. K. (2008). Distinct roles of Nox1 and Nox4 in basal and angiotensin II-stimulated superoxide and hydrogen peroxide production. *Free Radical Biology and Medicine*.

[13] Schramm A., Matusik P., Osmenda G., Guzik T. J. (2012). Targeting NADPH oxidases in vascular pharmacology. *Vascular Pharmacology*.

[14] Cohen R. A., Tong X. (2010). Vascular oxidative stress: the common link in hypertensive and diabetic vascular disease. *Journal of Cardiovascular Pharmacology*.

[15] Lassègue B., Griendling K. K. (2010). NADPH oxidases: functions and pathologies in the vasculature. *Arteriosclerosis, Thrombosis, and Vascular Biology*.

[16] Montezano A. C., Burger D., Ceravolo G. S., Yusuf H., Montero M., Touyz R. M. (2011). Novel nox homologues in the vasculature: focusing on Nox4 and Nox5. *Clinical Science*.

[17] Nowak T., Niemiec P., Żak I. (2013). Białko p22phox oraz gen CYBA. Ich funkcja i zwiazki z chorobami o podlozu miazdzycowym. *Wiadomosci Lekarskie*.

[18] Niemiec P., Nowak T., Iwanicki T. (2014). The 2930A>G polymorphism of the CYBA gene is associated with premature coronary artery disease. A case-control study and gene-risk factors interactions. *Molecular Biology Reports*.

[19] Niemiec P., Zak I., Wita K. (2007). The 242T variant of the CYBA gene polymorphism increases the risk of coronary artery disease associated with cigarette smoking and hypercholesterolemia. *Coronary Artery Disease*.

[20] Niemiec P., Nowak T., Balcerzyk A., Krauze J., Zak I. (2011). The CYBA gene A640G polymorphism influences predispositions to coronary artery disease through interactions with cigarette smoking and hypercholesterolemia. *Biomarkers*.

[21] Friedewald W. T., Levy R. I., Fredrickson D. S. (1972). Estimation of the concentration of low-density lipoprotein cholesterol in plasma, without use of the preparative ultracentrifuge. *Clinical Chemistry*.

[22] Khoury M. J., Flanders W. D. (1996). Nontraditional epidemiologic approaches in the analysis of gene- environment interaction: case-control studies with no controls!. *American Journal of Epidemiology*.

[23] Cortina-Borja M., Smith A. D., Combarros O., Lehmann D. J. (2009). The synergy factor: a statistic to measure interactions in complex diseases. *BMC Research Notes*.

[24] Datla S. R., Griendling K. K. (2010). Reactive oxygen species, NADPH oxidases, and hypertension. *Hypertension*.

[25] Li J.-M., Gall N. P., Grieve D. J., Chen M., Shah A. M. (2002). Activation of NADPH oxidase during progression of cardiac hypertrophy to failure. *Hypertension*.

[26] Byrne J. A., Grieve D. J., Bendall J. K. (2003). Contrasting roles of NADPH oxidase isoforms in pressure-overload versus angiotensin II-induced cardiac hypertrophy. *Circulation Research*.

[27] San José G., Moreno M. U., Oliván S. (2004). Functional effect of the p22phox -930A/G polymorphism on p22phox expression and NADPH oxidase activity in hypertension. *Hypertension*.

[28] Moreno M. U., José G. S., Fortuño A., Beloqui Ó., Díez J., Zalba G. (2006). The C242T CYBA polymorphism of NADPH oxidase is associated with essential hypertension. *Journal of Hypertension*.

[29] Schreiber R., Ferreira-Sae M. C., Ronchi J. A. (2011). The C242T polymorphism of the p22-phox gene (CYBA) is associated with higher left ventricular mass in Brazilian hypertensive patients. *BMC Medical Genetics*.

[30] Fan K.-L., Zhang H.-F., Zhu Z.-Y. (2013). Association of CYBA rs7195830 polymorphism with estimated glomerular filtration rate in an adult Han sample from Jiangsu province, China. *Chinese Medical Journal*.

[31] Liu C., Rennie W. A., Carmack C. S. (2014). Effects of genetic variations on microRNA: target interactions. *Nucleic Acids Research*.

[32] Zhu Z., Zhang H., Yao W. (2012). Physical activity modifies the association between CYBA gene polymorphisms and small artery elasticity in a Chinese population. *Hypertension Research*.

[33] Wang S. S., Bratti M. C., Rodríguez A. C. (2009). Common variants in immune and DNA repair genes and risk for human papillomavirus persistence and progression to cervical cancer. *Journal of Infectious Diseases*.

[34] Li P., Qiu T., Qin C. (2015). NADPH oxidase p22phox C242T polymorphism and ischemic cerebrovascular disease: an updated meta-analysis. *Medical Science Monitor*.

[35] Wu Z., Lou Y., Jin W. (2013). Relationship of the p22phox (CYBA) gene polymorphism C242T with risk of coronary artery disease: a meta-analysis. *PLoS ONE*.

[36] Hu P., Huang M.-Y., Hu X.-Y. (2015). Meta-analysis of C242T polymorphism in CYBA genes: risk of acute coronary syndrome is lower in Asians but not in Caucasians. *Journal of Zhejiang University SCIENCE B*.

[37] Xu Q., Yuan F., Shen X. (2014). Polymorphisms of C242T and A640G in CYBA gene and the risk of coronary artery disease: a meta-analysis. *PLoS ONE*.

